# High Purity Heroin Use Among Women in Karaj, Iran: A Pilot Study

**Published:** 2013

**Authors:** Masuadeh Babakhanian, Sahar Mansouri, Zahra Moradi, Zahra Abarashi

**Affiliations:** 1 Department of Social Work, Velayat Hospital, Semnan University of Medical Sciences, Damghan, Iran.; 2 PhD Student, Department of Psychology, Islamic Azad University, Karaj Branch, Karaj, Iran.; 3 Clinical Psychologist, Psychological Services Centre, Alzahra University, Tehran, Iran.; 4 Clinical Psychologist, Psychological Services Centre, Islamic Azad University, Karaj Branch, Karaj, Iran.

**Keywords:** Etiology, High Purity Heroin, Treatment, Women

## Abstract

**Objective:** High purity heroin use is a new epidemic health concern among Iranian drug-using women in recent years. However, the nature and initial factors associated with this serious health problem have not been studied yet.

**Methods:** To understand this issue, a cross-sectional study was conducted and sixty treatment and non-treatment seekers who met the Diagnostic and Statistical Manual of Mental Disorders, 4^th^ ed., Text Revision (DSM.IV-TR) criteria for drug dependence with mean age of 28.7 (± 8.3) years were recruited from 16 addiction clinics and drop-in centers (DICs) in Karaj, Iran. First, demographics, and details of drug use and risky behaviors were collected based on items elicited from Addiction Severity Index (ASI). Then, initial factors associated with high purity heroin use were collected by interviewing and applying a researcher-made questionnaire. Data was statistically analyzed by descriptive methods of statistics, chi-square, and Student’s independent t-test in SPSS software.

**Results: **Initiation of high purity heroin use was facilitated by family/relatives factor (66.7%), curiosity (60.1%), peers (54%), desire to experience rapid physical effects of high purity heroin compared with other opioids (50.1%), and treatment of general medical problems such as colic pain (33%). Chi-square test showed that non-treatment seekers were younger (less than 35 years) (66.6% vs. 56%, p ≤ 0.001) and mostly living with drug-dependent friends (26% vs. 3.3%, p ≤ 0.001) compared with treatment seekers. No relation was found among other factors.

**Conclusions:** Factors facilitating the initiation of high purity heroin use revealed in this study have not been fully addressed by current drug treatment services in Iran. To design comprehensive treatment programs, further studies on larger samples with more women are necessary.

**Declaration of interest:** None.

## Introduction

For centuries, Iranians were traditional users of opium. However, in recent years, opium use has been increasingly substituted for high purity heroin use (colloquially named heroin Kerack in Iran). The underlying reasons associated with this problem have not been studied yet. High purity heroin or heroin Kerack is a prevalent type of purified heroin in Iran. There is no comprehensive nationwide survey on the prevalence of high purity heroin use among Iranian women. However, a study shows that there are about 100,000 to 150,000 female opioid addicts, and high purity heroin abuse is a serious problem among women (1). Several studies have evaluated factors that facilitate the initiation of drug use in Iran. However, the majority of these studies have not specifically focused on women and high purity heroin use. This is due to high purity heroin being new in Iran and the desire of women to hide their addiction to drugs. For example, a study on 501 Iranian university students (172 women, and 329 men) showed that drug use was more prevalent among male participants compared with female participants. Moreover, pleasure seeking and modeling were the most important reasons for initial drug use, but seeking pleasure and relieving tension were also commonly reported reasons for recent drug use (2).

Another research studied the prevalence and predisposing factors associated with opioid use among 480 patients with the problem of chronic pain (of whom 57.5% were female) in Zahedan, Iran. In this study, no relationship was found between opioid use and chronic pain. However, there was a relationship between previous opioid use by friends, job, cigarette smoking, consultation for a psychological problem, and death of a spouse (1). Dolan et al. studied 97 addicted women in a methadone treatment clinic in Tehran, Iran. They revealed that more than 70% of the women reported high purity heroin as their current drug and smoking as their common route of use (3). In a study on socio-cultural factors associated with the initiation of opium use in Darab, Iran, Jafari et al. interviewed 76 drug users, aged 20-43, of whom 95% were men, and only 5% were women. The study findings indicated that the lack of recreational activities, treatment of general medical problems, peer pressure, friend networks, and cultural norms were the most important factors that facilitated the initiation of opium use. Moreover, partner pressure was an important factor among female participants (4). There is a serious paucity of research on female high purity heroin users in Iran, while there is evidence that high purity heroin dependence is a newly epidemic problem among women in large cities, such as Karaj. To our knowledge, no study has been conducted on factors facilitating the initiation of high purity heroin use among females in Karaj. The main aim of the current introductory study was to explore factors that facilitated the first-time high purity heroin use in a sample of female treatment seekers who were in methadone maintenance treatment in Karaj.

## Materials and Methods

Women aged 18-65 years who met the DSM.IV-TR (Diagnostic and Statistical Manual of Mental Disorders, 4^th^ ed., Text Revision) criteria for heroin dependence within the past twelve months were eligible to enter the study. The aim of the study was to conduct an introductory study on initial factors associated with high purity heroin use in a sample of women treatment and non-treatment seekers in Karaj, Iran. We recruited the sample through poster demonstrations in nearly all treatment centers, but only thirteen clinics and three drop-in centers (DICs) had women patients and agreed to cooperate with us. Inclusion criteria included being eighteen years of age or older, being a chronic high purity heroin abuser for at least twelve months prior to current treatment entry for addiction, signing a consent form for study participation, being in treatment for high purity heroin abuse, and living in Karaj during the time of the study. Exclusion criteria included presence of intoxication and withdrawal symptoms and signs, self-report of severe depression and suicidal thoughts which could interfere with the study procedure. Of the 72 women, 60 women met our study inclusion criteria, and were included in the study and interviewed. All participants provided a written consent form for study participation. 

*Study Design*

This was a cross-sectional and descriptive study and aimed to preliminarily explore the general descriptive profile of high purity heroin use problem among female treatment seekers and non-treatment seekers in Karaj. First, we recruited 30 treatment seekers and then asked them to bring with them female non-treatment seekers who they knew and were living in Karaj. Participation was voluntary and confidential. A consent form was obtained from each participant. Demographics, details of drug use, and risky behaviors were collected based on items elicited from Addiction Severity Index (ASI), as a highly valid international instrument. Then a confidential interviewing was conducted with the help of a researcher-made questionnaire to explore factors that facilitated initial factors associated with starting high purity heroin use. A pretest-posttest within two weeks on 20 women confirmed the high reliability of the questionnaire (Cronbach’s alpha: 96%) to meet the study aims. The validity of the questionnaire was evaluated by reviewing previous similar studies, and consulting with senior scholars and researchers in this field (3, 4). All study procedures were conducted in a quiet neutral interview room in each center. 

*Data Analysis*

Quantitative data including demographics, and details of drug use and risky behaviors were analyzed by descriptive methods of statistics, chi-square test, and Student’s independent t-test analysis in SPSS for Windows (version18; SPSS Inc., Chicago, IL., USA).

## Results

The sample was comprised of 30 female treatment seekers and 30 non-treatment seekers between 18 to 55 years of age [mean age 28.7 ± 11.3) years]. The majority of participants were married (36.6%), but we also interviewed women who were not currently married. Most of them were homemakers (56.6%), unemployed (16.6%), and were living with their families (78.3%). The mean age of the sample was 28.7 (± 8.3) years at the time of high purity heroin initiation, and the duration of dependency to high purity heroin use was 8 (± 9.4) years.

The general characteristics of participants and details of high purity heroin use among them are presented in table 1.

Data analysis by performing chi-square test showed that non-treatment seekers were younger (less than 35 years) (66.6% vs. 56%, p ≤ 0.001) and living more with drug-dependent friends (26% vs. 3.3%, p ≤ 0.001) compared with treatment seekers.

Table 2 shows the profile of risky behaviors among participants. A few participants reported condom use. As table 2 shows only 36.7% reported that they used condom during sex. 48.4% of them had sex with others (e.g. had sex throughout their lives with different partners). 31.7% of them had sex throughout their lives for drugs. 93.4% of them had no history of injection. 31.7% had a history of arrest and imprisonment. The majority of interviewed participants (66.7%) reported that a high purity heroin-using family member was an important factor in first-time high purity heroin use. 60.1% reported that they were curious to know what high purity heroin was. In 54% of them, the first experience with high purity heroin use was associated with recommendations of friends. Some of the participants stated that they relied on their addicted friends and used high purity heroin with them. 50.1% reported that they referred to high purity heroin as a new drug with rapid addictive effects on their bodies. Many women in the study reported that heroin was introduced to them as an opioid with strong physical effects. 33% reported that they smoked high purity heroin as a treatment, or adjunct to treatment of chronic conditions such as colic pain, and insomnia. 

**Table 1:** Demographics and details of high purity heroin in the two groups

**Variable**		**Treatment seekers** **(n = 30)**	**Non-treatment seekers** **(n = 30)**	**Overall sample** **(n = 60)**	**P-value**
**Age (%)**	**< 35 years**	17 (56.0)	20 (66.6)	37 (61.6)	< 0.01
	**> 35 years**	13 (44.0)	10 (33.4)	23 (38.3)	0.13
**Mean age (Year)**		28.7 (± 10.3)	27.7 (± 12.6)	28.7 (± 11.3)	0.12
**Marital status (%)**	**Married**	10 (33.0)	12 (40.0)	22 (36.6)	0.17
	**Single**	10 (33.0)	10 (33.0)	20 (33.3)	0.23
	**Divorced**	5 (16.6)	3 (10.0)	8 (13.3)	0.18
	**Separated**	4 (13.3)	3 (10.0)	7 (11.6)	0.34
	**Widow**	1 (3.3)	2 (6.6)	3 (5.0)	0.15
**Educational status (%)**	**< 12 years**	20 (66.6)	17 (56.6)	37 (61.6)	0.23
	**12 years**	6 (20.0)	7 (23.3)	13 (21.6)	0.34
	**> 12 years**	5 (16.6)	5 (16.6)	10 (16.6)	0.45
**Average education (Year)**		10.4 (± 8.7)	10.5 (± 4.7)	10.3 (± 5.9)	0.34
					
**Living condition (%)**	**With family**	23 (76.6)	24 (80.0)	47 (78.3)	0.34
	**With friends**	4 (13.3)	5 (16.6)	9 (10.0)	0.23
	**No definite place**	2 (6.6)	2 (6.6)	4 (6.6)	0.34
**Employment (%)**	**Homemaker**	16 (53.3)	17 (56.6)	34 (56.6)	0.23
	**Unemployed**	7 (2.3)	6 (0.2)	13 (21.6)	0.12
	**Employed**	3 (0.1)	4 (13.3)	7 (16.6)	0.13
	**Student**	3 (0.1)	3 (0.1)	6 (1.0)	0.80
**Onset age of high purity heroin use (Year)**		23 (± 10.2)	24 (± 8.1)	24 (± 9.1)	0.12
**Age of dependency on high purity heroin (Year)**		26 (± 7.8)	25 (± 9.7)	25 (± 6.8)	0.23
**Duration of dependency to high purity heroin use (Year)**		8.4 (± 8.3)	7.9 (± 10.4)	8 (± 9.4)	0.15
**Money spent on high purity heroin use (Last month in Rials)**		2090	2080	2080	0.123
**Current living with drug-dependent friends**		1 (3.3)	8 (26.0)	9 (15.0)	< 0.01

**Table2:** Risky behaviors among participants

**Variable**	**Treatment seekers (%)** **(n = 30)**	**Non-treatment seekers (%)** **(n = 30)**	**Overall sample (%)** **(n = 60)**	**P-value**
**Condom use**	10 (33.0)	12 (40.0)	22 (36.7)	0.12
**Sex with others**	15 (50.0)	14 (46.6)	29 (48.4)	0.16
**Sex for drugs**	9 (30.0)	10 (33.0)	19 (31.7)	0.23
**History of drug injection**	1 (3.3)	1 (3.3)	2 (6.6)	0.12
**History of arrest and imprisonment**	10 (33.0)	9 (30.0)	19 (31.7)	0.33
**A first degree relative or a close friend Infected with HIV**	1 (0.3)	1 (0.3)	2 (3.4)	0.13

## Discussion

The present pilot study is the first study in Iran to explore factors related to initiation of high purity heroin use in a sample of females in Karaj, Iran. This study may help policy makers target gender-specific needs when designing treatment services for women in Karaj. 

One important finding of our study was the relatively young age of initiation of high purity heroin use among the sample; this is in line with findings of studies from other parts of Iran (5). This raises the need for community-level programs that target this population before high purity heroin initiation. It was reported by our respondents that their high purity heroin-using family members and relatives were the most important factors for initial high purity heroin use. In fact, high purity heroin was directly introduced to them by their addicted family members or relatives. This study finding is in agreement with some similar studies which show that women are more likely to be drug-addicted if they have relatives with drug use problem (6, 7). This finding supports a part of an Indian study which revealed that among a variety of critical incidents influencing first drug use, the prevalence of drug use among family members and family acceptance facilitated pathways of female participants to drug use (8). An Iranian study revealed that partner pressure was an important factor for first time drug use among women (4). 

This finding may be partly explained by the close relationships that participants had with their family members and relatives. On the other hand, because of the stigma attached to illicit drug use within the Iranian female population, as well as the traditional beliefs of the community that consider providing and smoking illicit drugs as a male-dominated activity, participants had to refer to their family members and relatives in providing and using high purity heroin. A substantial group of interviewees stated that curiosity was a facilitating factor for initial high purity heroin use. This issue may be partly explained by the introduction of high purity heroin as a new opioid to participants. A similar Iranian study on initial alcohol use in a sample of men and women in Shiraz, Iran, showed that curiosity was an important factor for initial alcohol use (9). Witteveen et al. studied factors associated with initiation of cocaine and heroin use in drug addicts in Amsterdam, Netherlands, and found that curiosity was an important facilitating factor (5).

Friends were revealed to play an important role in initiation, but this issue was less reported compared with the two first facilitating factors. This is probably because the majority of respondents were married women with high purity heroin-using family members. Therefore, they were more likely to be introduced to high purity heroin by their relatives rather than their friends. This study finding supports some studies from other provinces of Iran. For example, in his study of drug use among students, Ghanizadeh revealed that the source of opium, hashish, and alcohol in first-time drug use was friends and peer groups (10). 

Witteveen et al. showed that some heroin and cocaine addicts referred to drug use for its effects of relieving negative feelings and experiencing drug regulatory effects (5). Moreover, self-treatment for general medical conditions such as physical pain and sleep disturbance was also a facilitating factor for first-time high purity heroin use. They had referred to high purity heroin as an opioid with stronger physical and mental effects compared with other drugs. This problem motivated some of the participants to refer to high purity heroin as an effective opioid for self-treatment of their general medical conditions. This is in line with some studies in other countries which show that women are more likely to use drugs for alleviation of physical or emotional pain (11, 7). The study findings showed that non-treatment seekers were younger and were mostly living with drug-dependent friends compared with treatment seekers. This issue necessitates the encouragement of non-treatment seekers to come for treatment and to separate them from drug-using environments. A Mexican study showed that being less than 35 years of age and living without a stable place contribute to more drug use (12).

This study had several limitations which should be considered when interpreting its findings. Our study was a pilot study and our study sample was selected from Karaj. Therefore, the factors facilitating initiation of high purity heroin use may not be applicable to other cities of Iran. However, our study was one of the few studies that target drug use among females in Iran. In conclusion, this brief study identifies factors facilitating initiation of high purity heroin use in a group of females in Karaj, Iran. Addressing the initial motivations of these women to start high purity heroin use might help in designing treatment programs based on their needs, and characteristics with the aim of removing factors that facilitated initial use. This strategy has never been carried out for female high purity heroin addicts in Iran. Gender-specific treatment services must be implemented to help these women quit high purity heroin dependence.
